# Combination of losartan with pirfenidone: a protective anti-fibrotic against pulmonary fibrosis induced by bleomycin in rats

**DOI:** 10.1038/s41598-024-59395-8

**Published:** 2024-04-16

**Authors:** Arian Amirkhosravi, Maryamossadat Mirtajaddini Goki, Mahmoud Reza Heidari, Somayyeh Karami-Mohajeri, Maryam Iranpour, Maryam Torshabi, Mitra Mehrabani, Ali Mandegary, Mehrnaz Mehrabani

**Affiliations:** 1 Physiology Research Center, Institute of Neuropharmacology, Kerman University of Medical Sciences, Kerman, Iran; 2 Department of Toxicology and Pharmacology, Faculty of Pharmacy, Kerman University of Medical Sciences, Kerman, Iran; 3 Pharmaceutics Research Center, Institute of Neuropharmacology, Kerman University of Medical Sciences, Kerman, Iran; 4 Department of Pathology, Pathology and Stem Cell Research Center, Faculty of Medicine, Kerman University of Medical Sciences, Kerman, Iran; 5 Department of Dental Biomaterials, School of Dentistry, Shahid Beheshti University of Medical Sciences, Tehran, Iran; 6 Herbal and Traditional Medicines Research Center, Kerman University of Medical Sciences, Kerman, Iran; 7 Department of Toxicology and Pharmacology, Faculty of Pharmacy, Shahid Sadoughi University of Medical Sciences, Yazd, Iran

**Keywords:** Idiopathic pulmonary fibrosis, Oxidative stress, Combination therapy, Bleomycin, Pirfenidone, Losartan, Respiratory tract diseases, Transforming growth factor beta

## Abstract

Pirfenidone (PFD), one acceptable medication for treating idiopathic pulmonary fibrosis (IPF), is not well tolerated by patients at full doses. Hence, employing of some approaches such as combination therapy may be applicable for increasing therapeutic efficacy of PFD. Losartan (LOS), an angiotensin II receptor antagonist, could be a suitable candidate for combination therapy because of its stabilizing effect on the pulmonary function of IPF patients. Therefore, this study aimed to investigate the effects of LOS in combination with PFD on bleomycin (BLM)-induced lung fibrosis in rats. BLM-exposed rats were treated with LOS alone or in combination with PFD. The edema, pathological changes, level of transforming growth factor-β (TGF-β1), collagen content, and oxidative stress parameters were assessed in the lung tissues. Following BLM exposure, the inflammatory response, collagen levels, and antioxidant markers in rat lung tissues were significantly improved by PFD, and these effects were improved by combination with LOS. The findings of this in vivo study suggest that the combined administration of PFD and LOS may provide more potent protection against IPF than single therapy through boosting its anti-inflammatory, anti-fibrotic, and anti-oxidant effects. These results hold promise in developing a more effective therapeutic strategy for treating of lung fibrosis.

## Introduction

Idiopathic pulmonary fibrosis (IPF) is an irreversible, chronic, fibroproliferative process and the most prevalent interstitial lung disease (ILD) of unknown etiology, leading to the decline of lung function^1^. A diagnosis of IPF is made by excluding other causes of interstitial pneumonia based on radiographic and histopathological findings^2^. IPF is characterized by pathologic hallmarks, including fibroblast proliferation, excessive extracellular matrix (ECM) production, collagen accumulation, and obstruction of the alveoli and airspaces, causing dyspnea^3^. The survival time is about four years post-diagnosis. The annual prevalence of IPF is between 3 and 9 cases per 100,000 individuals in North America and Europe^4^. Susceptibility to pulmonary fibrosis (PF) after COVID-19 recovery could be remarkable^5^. There is no exact information about the pathomechanisms of IPF. However, it may be caused by repeated local injuries of the alveolar epithelium and overproduction of reactive oxygen species (ROS)^6^. The produced ROS promoted the pro-fibrotic mediators such as transforming growth factor-β (TGF-β), inducing myofibroblast differentiation and ECM synthesis as the central role in developing IPF. TGF-β1 belongs to the TGF-β superfamily, a potent inducer of collagen type I and α-smooth muscle actin (α-SMA) expression that accelerates PF progression^7^. Hence, reducing oxidative stress and inhibiting the TGF-β1 production might be a practical therapeutic approach for attenuating PF process.

In 2014, the U.S. Food and Drug Administration approved pirfenidone (5-methyl-1-phenyl-2-(1 H)-pyridone, PFD) for treating IPF. PFD exhibited an anti-fibrotic effect by decreasing pro-fibrotic cytokines (like TGF-β1) and anti-proliferative and anti-oxidant features, which reduce progression of IPF via several mechanisms. These mechanisms include attenuating epithelial-mesenchymal transition (EMT), collagen deposition, and fibroblast proliferation^8–10^. More importantly, this drug is widely used to treat the global SARS-CoV-2 pandemic^5^. Despite the acceptable anti-fibrotic effects of PFD, neither the patient tolerates the full dose of the drug nor does it improve the survival rate of the patients over 2 years^11^. Hence, ongoing efforts have been made to explore a new therapeutic strategy or an alternative therapy for IPF.

The use of combination medication therapy shows promise as a treatment option for this disease with a high mortality rate. There are several advantages, such as reducing individual drug doses, minimizing side effects, achieving multiple complementary therapeutic goals, and decreasing the risk of resistance^12^. In our previous study, we showed combination therapy with PFD and prednisolone had more potent effects in paraquat (PQ)-induced lung fibrosis in rats^13^. Losartan (LOS), an angiotensin II receptor antagonist, maybe a good candidate for the combination therapy due to its stabilizing effect on the lung function of IPF patients besides its relatively low toxicity profile^14^. Furthermore, during IPF, angiotensin II can increase TGF-β production; hence it is reasonable that blocking the receptor of angiotensin II by LOS can delay the progression of IPF^15^. In addition to scavenging ROS, LOS also decreases activation of the pro-oxidative/inflammatory pathways^16^.

Bleomycin (BLM) is a classic experimental model of IPF in rats. BLM, a standard chemotherapeutic agent, is used in several malignancies. However, the primary hurdle for its clinical usage is the induction of PF caused by a deficiency of BLM hydrolase enzyme activity in lung tissue. The results are the induction of oxidative stress and DNA strand breakage of lung cells. Due to these side effects, BLM is commonly used to induce an animal model of IPF^17^. Based our previous in vitro study, combination of PFD with LOS showed a greater efficacy in regulating the epithelial–mesenchymal transition (EMT) process and oxidative stress in human A549 cells than single therapy^18^. Therefore, to complete our work, we tested the efficacy of LOS in combination with PFD for moderating BLM-induced lung fibrosis in rats by measuring the histological, inflammatory, hydroxyproline (HYP), and oxidative markers.

## Materials and methods

### Drugs and chemicals

BLM sulfate was obtained from Selleck Chemicals, Houston, TX, USA, and PFD from Intermune Company, USA. LOS, HYP (Cat No: MAK463), and all histological staining reagents (Cat No: GHS332, R03040, HT15) were acquired from Sigma‐Aldrich Co. Ltd., St. Louis, MO, USA. The TGF-β1 kit (Cat No: DB100B) was purchased from R&D Systems, USA. Catalase (CAT), superoxide dismutase (SOD), and malondialdehyde (MDA) assay kits were purchased from Teb Pazhouhan Razi (TPR), Tehran, IRAN.

### Animals

Fifty-six adult male *Wistar* rats (average weight 200–250 g, 6–8 weeks old ) were obtained from the Kerman University of Medical Sciences animal center and maintained under regulated conditions as explained in the previous investigation^9^. Approval for the study was granted by the Ethics Committee of Kerman University of Medical Sciences (IR.KMU.REC.1398.422) and followed by the Animal Research: Reporting of In Vivo Experiments (ARRIVE) guidelines. The methods were performed in accordance with these relevant guidelines and regulations by including a statement in the methods section to this effect.

### Experimental design and BLM-induced pulmonary fibrosis

**Figure 1:**
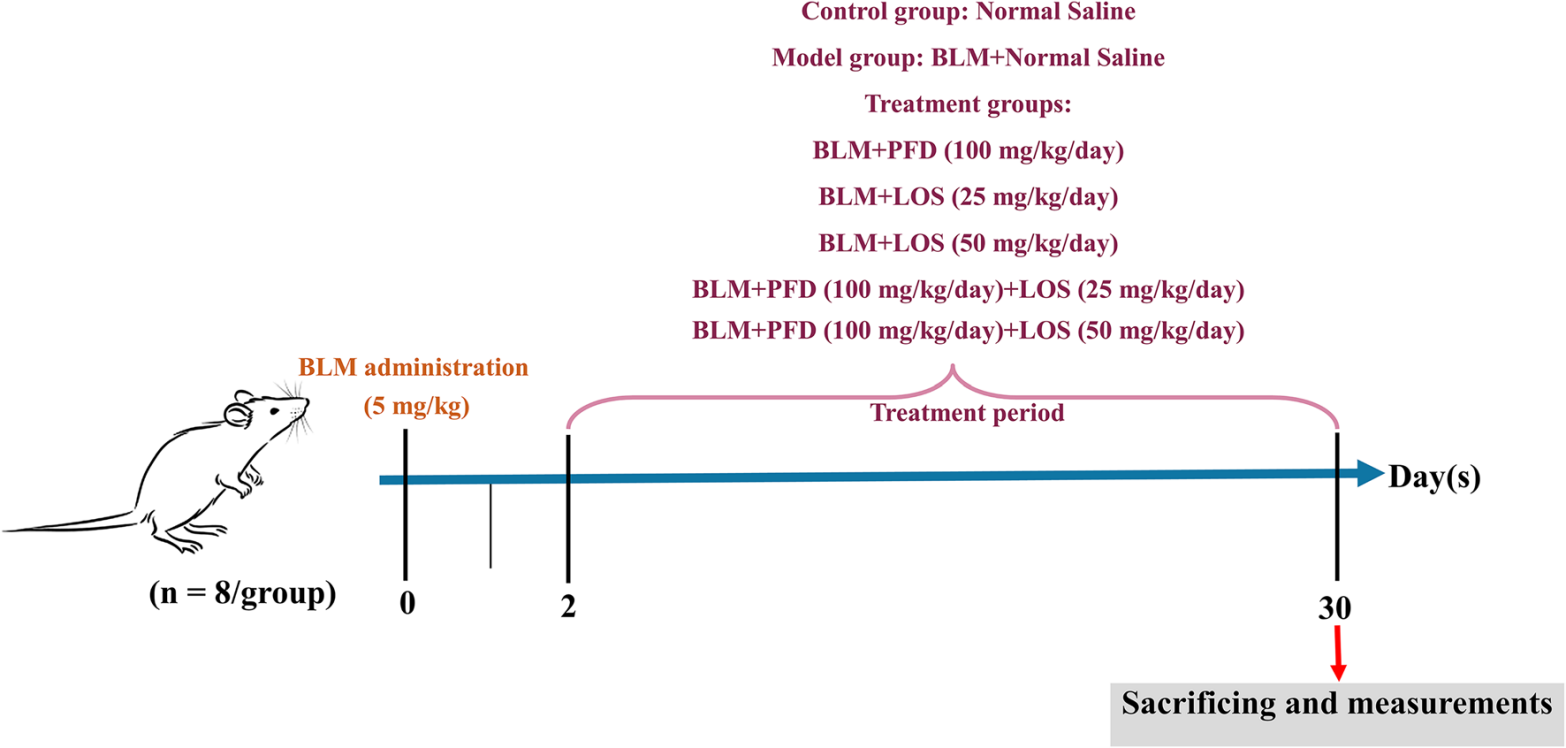
The experimental design and study timeline for bleomycin (BLM) administration and treatment protocol in different rat groups. Induction of IPF model with BLM on day 0 and treatment with LOS, PFD, and their combination two days after bleomycin induction for 28 consecutive days. Animals were sacrificed on day 30. *BLM* bleomycin, *LOS* losartan, *PFD* pirfenidone, *IPF* idiopathic pulmonary fibrosis.

Before the randomization of the animals, the animals’ skin was shaved at the desired location and their total body weight was measured. Next, rats were randomly grouped into seven, as presented in Fig. 1 they were anesthetized intraperitoneally (i.p.) with ketamine/xylazine (80/8 mg/kg) before BLM injection based on the previous study^19^. BLM-induced IPF model received one dose of bleomycin sulfate (5 mg/kg) intratracheally (i.t.) on day 0^8^, followed by oral normal saline administration; the BLM + PFD group received 5 mg/kg bleomycin (day 0) then 100 mg/kg/day pirfenidone as suggested in the previous study^9^; BLM + LOS groups received 5 mg/kg bleomycin (day 0) then 25 and 50 mg/kg/day losartan; BLM + PFD + LOS groups received 5 mg/kg bleomycin (day 0) then combinations of 25 mg/kg/day losartan + 100 mg/kg/day pirfenidone or 50 mg/kg/day losartan + 100 mg/kg/day pirfenidone. All the treatments were administered via gavage two days after bleomycin induction for 28 consecutive days^8^. The animals in the control and BLM groups were administered with the same saline solution as a carrier for LOS and PFD. Body weights were monitored daily.

### Collection of lung tissues and bronchoalveolar lavage fluid (BALF)

Upon completion of the treatment period (day 30), the animals were sacrificed with a high-dose injection of ketamine/xylazine through i.p. injection. Lungs were divided into several halves. The section of left lung was snap frozen in liquid nitrogen and stored at − 70 °C for analysis of oxidative stress and hydroxyproline content. The remaining portion of the left lung was immersed in 10% buffered formalin for histopathological examination. The right lungs were weighed to determine their dry weight. For BALF collection, the trachea was cannulated and lavaged with 1 mL of normal saline twice, then aspirated normal saline at 37 °C as explained previously^9^. Subsequently, the lungs were extracted and weighed. The left lungs were preserved in 10% formalin and embedded in paraffin for histopathological examination. The right lungs tissues were excised for other parameters analysis. To evaluate the protein content of the lungs, phosphate buffer was used to homogenize all samples. The Bradford method was used to determination of protein concentration of samples^20^.

### Measurement of lung weight

To determine pulmonary edema, lung tissues were washed with isotonic saline and their wet weight was measured. After being incubated at 60 °C for 72 h, the lungs were weighed to determine their dry weight. The lung wet/dry (W/D) weight ratio was evaluated by dividing the wet weight by the dry weight of the same lung^21^.

### Measurement of the histopathological marker

Fixed section of left lungs was embedded in paraffin, sectioned (5 μm), stained using hematoxylin–eosin (H&E), Masson’s trichrome, and viewed through a light microscope at 100X magnification (Olympus BX53, Japan) for determination of histopathological analysis. The samples blindly were analyzed and scored by the pathologist (6 random fields/animals) for the degree of fibrosis, congestion of red blood cells (RBCs), and pneumocyte hyperplasia on subjective categories of none (0), patchy changes (1), local changes (2), scattered changes (3), severe changes (4) and the averages were considered. The sections were also subjected to quantify the collagen fiber deposition based on Masson’s trichrome staining, according to the earlier report^21^.

### Measurement of TGF-β1 in BALF

The concentration of TGF-β1 in the BALF was measured using a commercially available enzyme-linked immunosorbent assay (ELISA) kit following the guidelines provided by the manufacturer.

### Measurement of HYP

A colorimetric evaluation was determined in the lung tissue to assess collagen content by measuring HYP. Briefly, 40 mg of lung tissues were homogenized while on ice and incubated with cupric sulfate, sodium hydroxide, and hydrogen peroxide at 80 °C for 5 min and then were chilled. Next, the samples were incubated with sulfuric acid and ρ-dimethylamino benzaldehyde in 1-propanol (80 °C for 30 min). The wavelength used to measure the absorbance value was 560 nm^22^. Furthermore, the HYP content in lung tissue was calculated using the HYP standard curve (µg/mg wet tissue).

### Measurement of malondialdehyde (MDA) and antioxidant enzymes

Based on the previous study, commercial kits were used to measure MDA as well as antioxidant markers in the supernatant of lung tissue^20^. The absorbance of samples was read at 532 nm (for MDA), 450 nm (for SOD activity), and 240 nm (for CAT activity) using a microplate reader (BioTek Instruments, Inc, Winooski, VT, USA).

### Statistical analysis

In order to check the normality distribution of the data, we performed the Kolmogorov–Smirnov test. For body weight data, differences between groups were performed through the one-way analysis of variance (ANOVA) using GraphPad Prism software version 8 (GraphPad Software Inc., San Diego, CA, USA). The data were analyzed from at least three independent experiments and expressed as the mean ± standard deviation (SD). To analyze the other tests, we performed the Kruskal–Wallis. Median and interquartile range was applied to the charts. *P* values ≤ 0.05 were considered statistically significant.

## Results

### Effect of PFD and LOS alone and their combination on the body weight and lung W/D weight rats exposed to BLM

**Figure 2:**
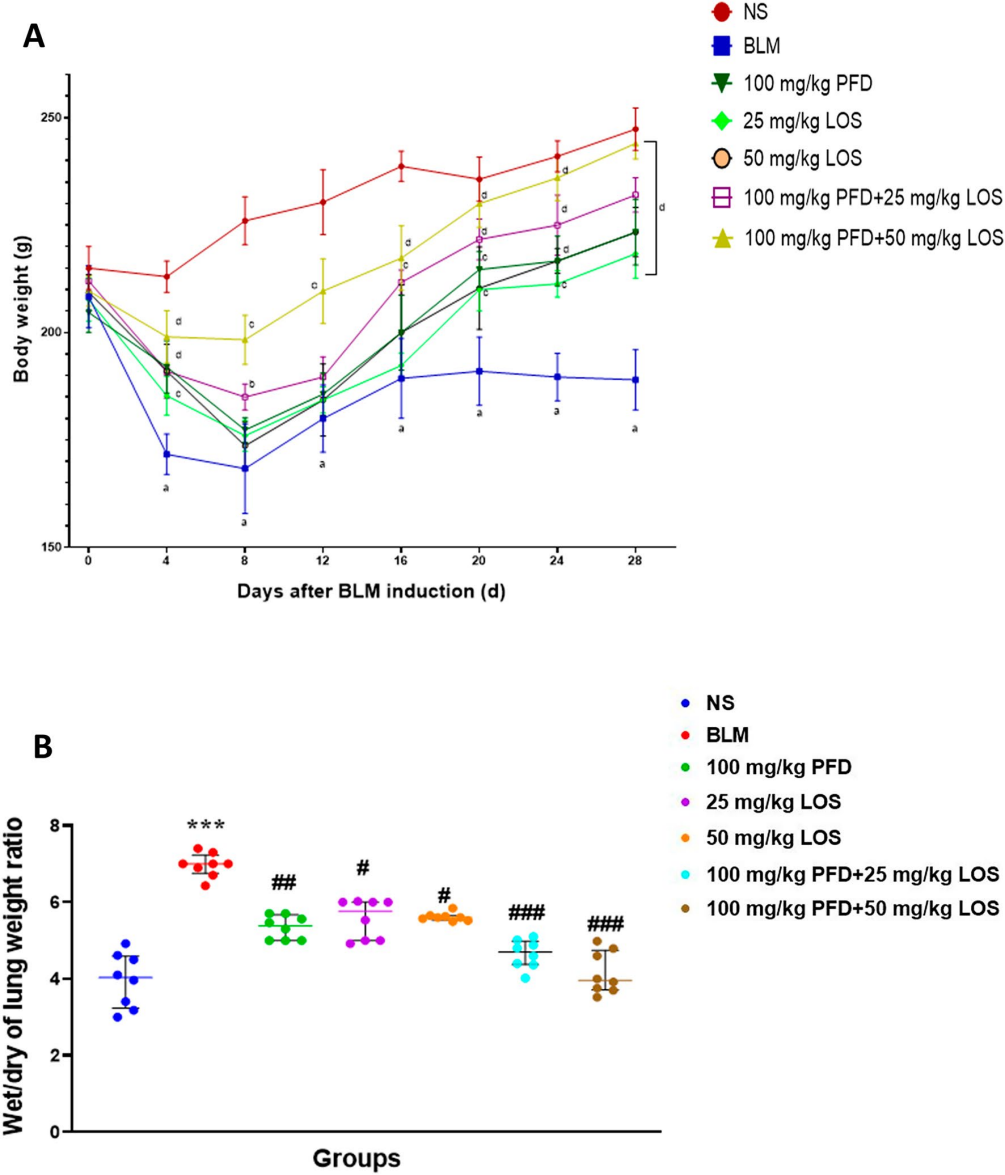
Effect of pirfenidone (PFD) and losartan (LOS) alone and combination of pirfenidone with losartan (PFD + LOS) on the rats exposed to bleomycin (BLM). (**A**) The body weight changes curve of rat versus time, and (**B**) Comparisons of W/D weight ratio of the lung between the control group (NS), the group exposed to BLM at doses of 5mg/kg on day 0, groups exposed to 5mg/kg BLM and treated with 100 mg/kg PFD (BLM + PFD 100 mg/kg), 25 and 50 mg/kg LOS (BLM + LOS 25 mg/kg and LOS 50 mg/kg), 100 mg/kg PFD + 25 mg/kg LOS (BLM + PFD 100 mg/kg + LOS 25 mg/kg), and 100 mg/kg PFD + 50 mg/kg LOS (BLM + PFD 100 mg/kg + LOS 50 mg/kg) from day 2 to day 28. Data are presented as the means ± SD (n = 8 per group). a: *P* < 0.001 indicates significant differences as compared with the control group; b: *P* < 0.05, c: *P* < 0.01, and d: *P* < 0.001 vs. the BLM group. *P < 0.05, **P < 0.01, and ***P < 0.001 indicate significant differences as compared with the control group; ^#^*P* < 0.05, ^##^*P* < 0.01, and ^###^*P* < 0.001 vs. the BLM group. *BLM* bleomycin, *LOS* losartan, *PFD* pirfenidone, *NS* normal saline.

To determine the suppressive fibrosis effects of PFD and LOS and their combination, rats were given 5 mg/kg BLM to induce the lung fibrosis model. As shown in Fig. 2A, body weight measurement showed a progressive loss in BLM-exposed rats compared with the control group. All treated rats represented a significant and progressive increase in weight regain during 28 days of treatment. Next, the lung W/D weight ratio significantly increased after BLM administration compared to normal control rats. Increased W/D of lung weight ratio was significantly reduced in all treated groups compared with the BLM group and more pronounced alleviating effects were observed in the rats treated with BLM + 100 mg/kg PFD + 25 mg/kg LOS and BLM + 100 mg/kg PFD + 50 mg/kg LOS (Fig. 2B).

### Effect of PFD, LOS, and combination of PFD + LOS on lung macroscopy and histological changes in BLM-induced rats

**Figure 3:**
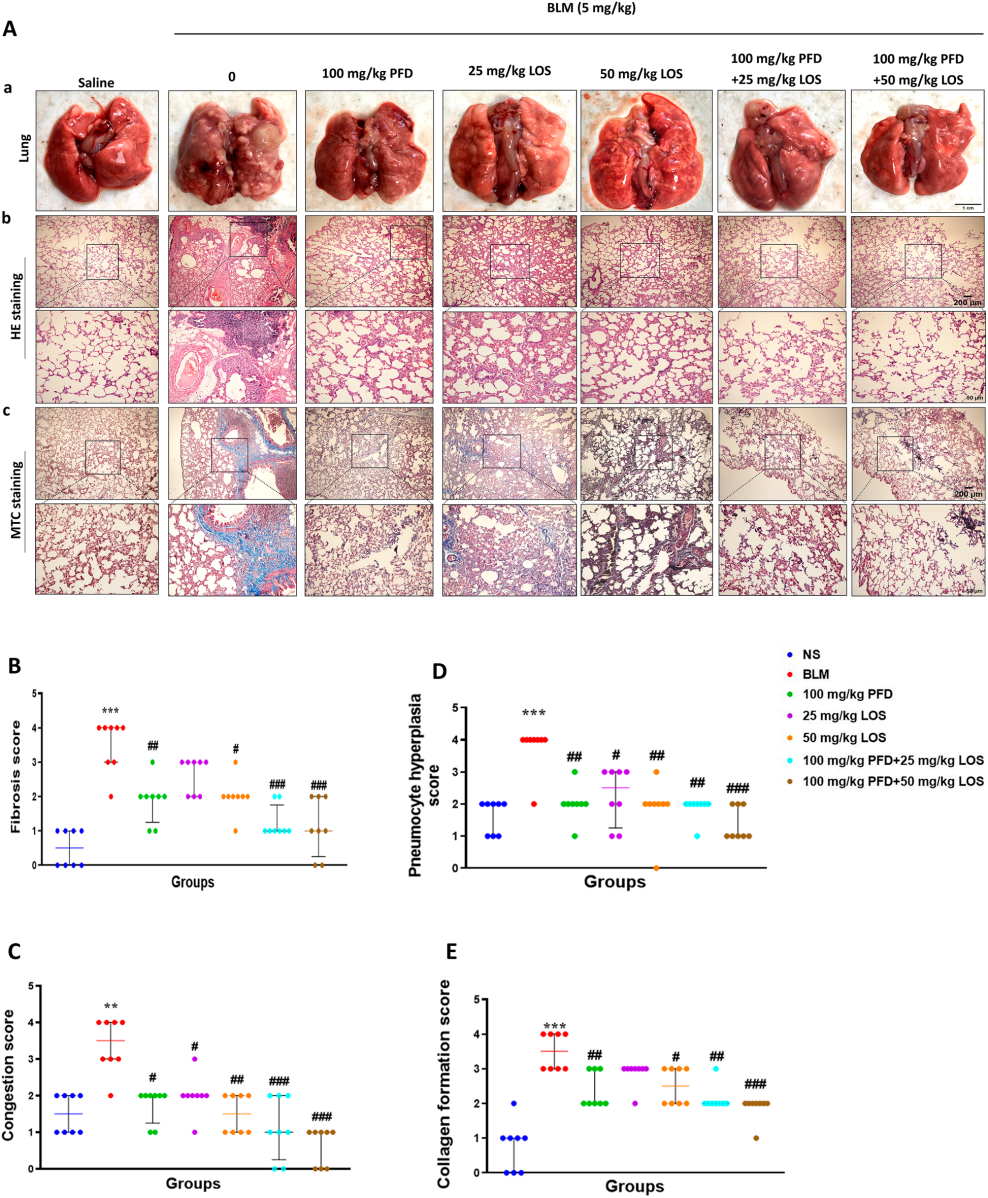
Effect of pirfenidone (PFD), losartan (LOS), and combination of pirfenidone with losartan (PFD + LOS) on macroscopic and microscopical changes stained with H&E (HE) and Masson’s trichrome (MTC). The rats were exposed to the BLM-induced pulmonary fibrosis model on day 0 (5mg/kg) and treated with 100 mg/kg PFD (BLM + PFD 100 mg/kg), 25 and 50 mg/kg LOS (BLM + LOS 25 mg/kg and LOS 50 mg/kg), 100 mg/kg PFD + 25 mg/kg LOS (BLM + PFD 100 mg/kg + LOS 25 mg/kg), and 100 mg/kg PFD + 50 mg/kg LOS (BLM + PFD 100 mg/kg + LOS 50 mg/kg) from day 2 to day 28. **(A)** Macroscopic observations and pathological microscopic changes in rat lung tissues after treatments; (**A**) Representative images depicted the appearance of the lungs across the experimental animal groups. Scale bar: 1 cm. (**B**) H&E staining, (**C**) Masson's trichrome staining of different groups. Magnification: X40 and X100; (**B**–**D**) Scoring of pulmonary fibrosis parameters; (**E**) Quantification of Masson's trichrome stained sections. **P < 0.01 and ***P < 0.001 vs. control group; ^#^*P* < 0.05, ^##^*P* < 0.01, and ^###^*P* < 0.001 vs. the BLM group. *BLM* bleomycin, *LOS* losartan, *PFD* pirfenidone, *NS* normal saline, *HE* hematoxylin–eosin stain, *MTC* Masson trichrome stain.

In the macroscopy images, the lungs of the control group appeared pink and dry, with no structural damage. However, the group that received BLM exhibited tissue discoloration, edema, and reduced elasticity, which were improved by all treatments (Fig. 3A). The control group showed typical lung tissue structure as revealed by H&E staining. The i.t. injection of BLM in the model group destroyed alveolar structures, thickening the alveolar septa, and severe fibrosis in lung tissue. Furthermore, pneumocyte hyperplasia and RBCs were significantly higher than the control group on day 28. While all the treatment groups exhibited a significant decrease in pneumocyte hyperplasia, RBCs accumulation, and fibrosis scores, all pathological lung changes significantly might be more protective by combination groups (BLM + 100 mg/kg PFD + 25 mg/kg LOS and BLM + 100 mg/kg PFD + 50 mg/kg LOS) compared with the BLM group (Fig. 3B–D). Concurrently, Masson trichrome staining of lung sections indicated abundant blue collagen deposition in BLM exposed group compared to the control group. However, the accumulation of collagen fibers significantly decreased in all treated groups, especially in the BLM + 100 mg/kg PFD + 25 mg/kg LOS and BLM + 100 mg/kg PFD + 50 mg/kg LOS treated groups, as compared to the BLM group (Fig. 3E). It seems that PFD + LOS combination is an effective therapy of BLM-induced lung fibrosis.

### Effect of PFD, LOS, and combination of PFD + LOS on BALF TGF-β1 levels and HYP content of the lung tissue exposed to BLM

**Figure 4:**
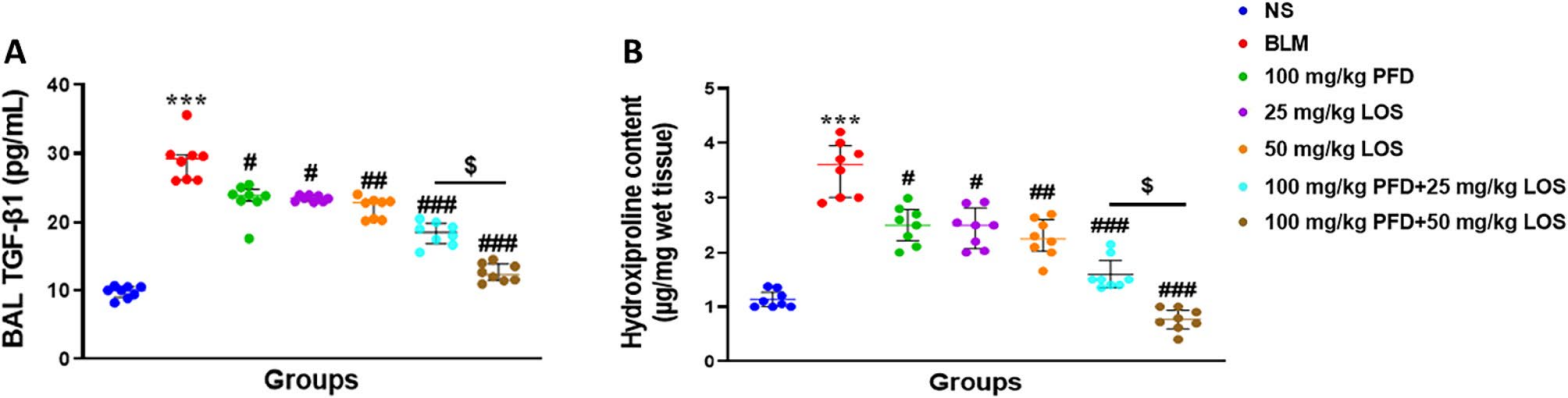
Effect of pirfenidone (PFD), losartan (LOS), and combination of pirfenidone with losartan (PFD + LOS) on bronchoalveolar lavage fluid transforming growth factor β1 (BALF TGF-β1) levels and hydroxyproline (HYP) content of the rats exposed to bleomycin (BLM). The rats were exposed to the BLM-induced pulmonary fibrosis model on day 0 (5mg/kg) and treated with 100 mg/kg PFD (BLM + PFD 100 mg/kg), 25 and 50 mg/kg LOS (BLM + LOS 25 mg/kg and LOS 50 mg/kg), 100 mg/kg PFD + 25 mg/kg LOS (BLM + PFD 100 mg/kg + LOS 25 mg/kg), and 100 mg/kg PFD + 50 mg/kg LOS (BLM + PFD 100 mg/kg + LOS 50 mg/kg) from day 2 to day 28. After that, BALF and lung tissue were collected. (**A**) ELISA quantified BALF TGF-β1 concentration and (**B**) HYP levels of lung tissue samples were expressed. *n* = 8 rats per group. ***P < 0.001 vs. control group; ^#^*P* < 0.05, ^##^*P* < 0.01, and ^###^*P* < 0.001 vs. the BLM group; ^$^*P* < 0.05 vs. PFD 100 mg/kg + LOS 25 mg/kg group. *BLM* bleomycin, *LOS* losartan, *PFD* pirfenidone, *NS* normal saline.

The TGF-β1 level in the BALF and HYP content in lung tissues were determined as shown in Fig. 4. TGF-β1 concentration and HYP content elevated significantly in the BLM-treated group compared with normal control rats. These changes were protected after all the treatments. In addition, combined drug treatment (100 mg/kg PFD + 25 mg/kg LOS and 100 mg/kg PFD + 50 mg/kg) was more effective in the reduction of TGF-β1 concentration and HYP content than the single ones compared with the BLM group. Furthermore, increasing the dosage of LOS in combination therapy with PFD significantly diminished the TGF-β1 concentration and HYP content after BLM delivery.

### Effect of PFD, LOS, and combination of PFD + LOS on oxidative stress parameters induced by BLM in the rats

**Figure 5:**
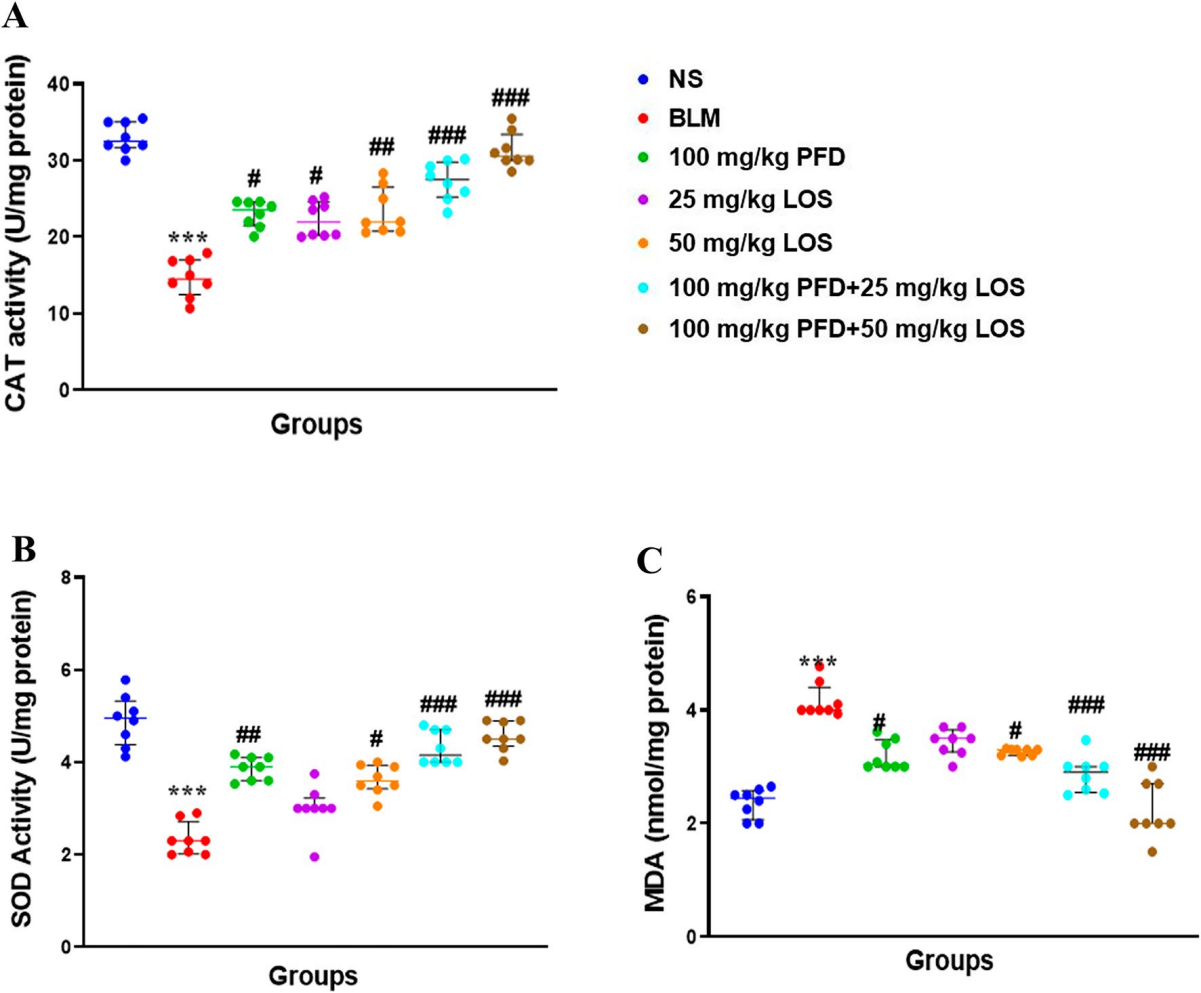
Effect of pirfenidone (PFD), losartan (LOS), and combination of pirfenidone with losartan (PFD + LOS) on oxidative stress parameters induced by bleomycin (BLM) in the rats. The rats received BLM (5mg/kg) on day 0 and treated with 100 mg/kg PFD (BLM + PFD 100 mg/kg), 25 and 50 mg/kg LOS (BLM + LOS 25 mg/kg and LOS 50 mg/kg), 100 mg/kg PFD + 25 mg/kg LOS (BLM + PFD 100 mg/kg + LOS 25 mg/kg), and 100 mg/kg PFD + 50 mg/kg LOS (BLM + PFD 100 mg/kg + LOS 50 mg/kg) from day 2 to day 28. Next, lung tissue was collected. (**A**) The CAT and (**B**) SOD enzymes activities and (**C**) MDA levels were determined in the lung of each group. *n* = 8 rats per group. ***P < 0.001 vs. control group; ^#^*P* < 0.05, ^##^*P* < 0.01, and ^###^*P* < 0.001 vs. the BLM group. *CAT* catalase, *SOD* superoxide dismutase, *MDA* malondialdehyde, *BLM* bleomycin, *LOS* losartan, *PFD* pirfenidone, *NS* normal saline.

To evaluate the supportive effect of LOS and PFD and their combination against oxidative stress, the CAT and SOD enzymes activity and the MDA content were examined. In the BLM group, a meaningful rise in MDA level and a decrease in the CAT and SOD enzymes activity was detected in lung tissue compared to the control group. As expected, all the treatments significantly mitigated the MDA level and restored the activities of CAT and SOD in the BLM-treated group compared to the control group. Considerably, the levels of oxidant and antioxidant biomarkers were significantly more normalized in the groups treated with 100 mg/kg PFD + 25 mg/kg LOS and 100 mg/kg PFD + 50 mg/kg doses compared with single treatments (Fig. 5).

## Discussion

IPF is a complex and fatal lung disease characterized by the abnormal accumulation of fibrotic tissue in the lung parenchyma. It has a high morbidity rate and a poor prognosis^6^. Significant progress has been made in understanding the pathophysiology of fibrosis and its treatment options. Here, breaking three substantial barriers were presented to the treatment of fibrosis: oxidative stress, inflammation, and fibrogenesis, which are all manifested in the present model of BLM-induced PF^9^.

As an anti-fibrotic compound, PFD is used to treat PQ-induced lung fibrosis in rats by inhibiting inflammation, oxidative stress, downregulating pro-fibrotic cytokines, and ROS generation enzymes^9^. Although this drug has great potential as an effective treatment for IPF, it is poorly tolerated by patients at the full dose^11,23^. Accordingly, there is an emerging need to find more effective therapeutic approaches for IPF patients. The combination therapy of PFD with vitamin E and prednisolone have good effects as a new therapeutic for anti-IPF^13,24^. We also worked on this idea to increase knowledge about combination therapy. An angiotensin II type 1 receptor antagonist, LOS, is commonly utilized to treat hypertension. It also has anti-fibrotic potential in the lung fibrosis model^15^. According our previous study, blocking the angiotensin II receptor can potentially decelerate the progression of IPF since angiotensin II is known to increase the levels of TGF-β in this condition^15^. LOS demonstrates significant inhibition of the TGF-β/Smad signaling pathway in lung fibrosis^25^. Also, LOS suppress the epithelial-mesenchymal transition (EMT) process and fibroblast activity^18^. Arterial hypertension is a comorbidity reported in patients with IPF causing disease progression and reduced quality of life. This leads to prescribing angiotensin II receptor blocker (ARB) drugs like LOS for many patients. ARBs modulate renin-angiotensin system activation and promote vasodilation. The fibrotic lung contains a high concentration of angiotensin peptides, and their activity can be manipulated to impact lung fibrosis in experimental models^26^. Angiotensin II can boost TGF-β production during IPF. Therefore, inhibiting the angiotensin II receptor with LOS could be a logical approach to slow down the progression of IPF^15^. Scavenging ROS and reducing inflammation can alleviate oxidative stress and lung inflammation^16^. Taken together, it appears that this combination is safe due to its stabilizing effect on the lung function of IPF patients, besides its relatively low toxicity profile^14^. Thus, the in vivo present study aimed to assess the protective effects of the LOS + PFD combination against BLM-induced lung fibrosis in rats for the first time.

As mentioned previously, BLM was employed as an inducer of oxidative stress to simulate the IPF model in vivo. After forming a complex with O_2_ and iron, BLM generates ROS, especially superoxide and hydroxyl radicals, that bind to the DNA helix leading to its breakage and subsequent oxidative events^20^. Our current study shows that the BLM significantly decreased SOD and CAT enzymes activity and enhanced MDA levels, an important marker of lipid peroxidation (LPO), compared to the control group. LOS and PFD increase the activation of SOD and CAT while decreasing MDA content in BLM-treated rats. However, when they were used in combination, their effects were more pronounced. Our result confirmed the previous study presenting that BLM can disturb the normal redox state of cells by decreasing the activity of antioxidant enzymes and increasing LPO^27^. Exposure to BLM increased angiotensin II in lung tissues. Angiotensin II can raise free radicals in liver fibrosis, renal injury, and myocardial infarction^28–30^. Guo et al. reported that angiotensin II type 1 receptor blockers had the potential to delay lung damage induced by free radicals through increased SOD levels while decreasing MDA contents^15^. Misra et al. found that PFD has an antioxidant activity by scavenging hydroxyl and superoxide anion free radicals. Furthermore, NADPH-dependent lipid peroxidation is blocked by PFD in sheep liver in a dose-dependent manner^31^. According to these results, LOS + PFD’s anti-fibrotic effect might be mediated by reducing ROS formation and oxidative stress.

In line with the findings of LPO and antioxidants, the H&E histopathological scoring and Masson’s trichrome observations of the lung sections revealed induction of fibrosis, congestion of RBCs, pneumocyte hyperplasia, and excessive accumulation of collagen as a hallmark for PF in the BLM group. Reactive type II pneumocyte hyperplasia refers to a non-specific reactive increase in the proliferation of type II pneumocytes, which occurs in PF^32^. BLM induces the activation of alveolar macrophages, leading to the production of inflammatory and profibrotic cytokines such as interleukin-1 (IL-1) and macrophage inflammatory protein-1. Nevertheless, BLM induces type II pneumocyte hyperplasia that unlike normal type II cells, contributes to the secretion of some of these cytokines. These cytokines further stimulate the proliferation and activation of fibroblasts, leading to increased collagen deposition^33^. Histological improvement in PFD, LOS, and combined LOS + PFD received groups confirmed the more potent anti-fibrotic effects of the LOS + PFD combination compared to single therapy. Our results agreed with previous literature showing the fibrotic effect of BLM in lung tissue^20,34^. It has been shown that PFD induces an anti-fibrotic impact on the PQ fibrosis model^9,13^. Moreover, LOS, through its potent antioxidant and anti-fibrotic potential, decreased lung fibrosis induced by BLM^28,35^. The results of our study about the type II pneumocyte hyperplasia are in line with Albanawany et al. who reported that decreased number of type II pneumocytes was evident in treatment groups^34,36^. In addition, PFD, LOS, and combined LOS + PFD reduced lung HYP levels caused by BLM. HYP is an amino acid that is produced during the biosynthesis of collagen. The extensive accumulation of extracellular matrix, such as collagen, in the alveoli is a characteristic feature of lung fibrosis. Thus, HYP is considered a critical diagnostic and the most objective method of fibrosis quantitation^37^. Different prior studies support the effect of BLM on the lung HYP contents, as seen in the current study^38–40^. Furthermore, it has been shown that PFD reduces lung HYP content in hamsters^41^. Also, LOS can inhibit the HYP contents in lung tissues^28^. Notably, the reduction in the level of fibrosis and collagen by combination therapy is significantly more than the reduction achieved by PFD or LOS as monotherapy.

Also, our study assessed pulmonary edema and lung inflammation by measuring the W/D weight ratio and TGF-β1 concentration. Increasing the W/D weight ratio and TGF-β1 concentration show that BLM augments lung inflammation and treatment with LOS, PFD, and combination of LOS with PFD inhibit this process. It was found that LOS and PFD, as a single treatment for IPF, can delay the progression of this disease. Interestingly, all changes were more marked in the combination group. These results support previous reports suggesting PFD’s deleterious effects in the lungs^24,42,43^. To inhibit PF, PFD alleviates macrophage-driven cytokines such as; Interleukin 1 beta (IL-1β), tumor necrosis factor-alpha (TNF-α), TGF-β1, and platelet-derived growth factor (PDGF)^44^. The promising anti‐inflammatory activity of ARBs has been reported in previous studies^35,45,46^.

It is recommended that future studies focus on the mechanism of the drugs and adverse effect of combination therapy. As both drug mechanisms are different, reduction of fibrosis using classic fibrosis markers can be studied.

## Conclusion

The results of our study demonstrated that LOS in combination with PFD increased the activation of SOD and CAT while decreasing MDA, HYP levels, W/D weight ratio, and TGF-β1 concentration also improved lung histological changes under BLM induction. The therapeutic implication of these conclusions suggests that the combined LOS + PFD could be beneficial for IPF patients. Our findings present promising data to support the possibility of clinical studies to confirm the effects of PFD in combination with LOS to treat IPF. This is the first study discovering the possibilities of using PFD with the LOS in vivo model. Nevertheless, future studies are needed to gather clarifying pre-clinical information supporting a clinical trial.

## Data Availability

All data generated or analyzed during this study are included in this manuscript. The data are not publicly available due to privacy or ethical restrictions.
